# Role of Zika Virus prM Protein in Viral Pathogenicity and Use in Vaccine Development

**DOI:** 10.3389/fmicb.2018.01797

**Published:** 2018-08-02

**Authors:** Peter Nambala, Wen-Chi Su

**Affiliations:** ^1^Graduate Institute of Biomedical Sciences, China Medical University, Taichung, Taiwan; ^2^Research Center for Emerging Viruses, China Medical University Hospital, Taichung, Taiwan

**Keywords:** ZIKV, prM, viral pathogenicity, vaccine, epidemic

## Abstract

Recent Zika virus (ZIKV) epidemics necessitate the urgent development of effective drugs and vaccines, which can be accelerated by an enhanced understanding of ZIKV biology. One of the ZIKV structural proteins, precursor membrane (prM), plays an important role in the assembly of mature virions through cleavage of prM into M protein. Recent studies have suggested that prM protein might be implicated in the neurovirulence of ZIKV. Most vaccines targeting ZIKV include prM as the immunogen. Here, we review progress in our understanding of ZIKV prM protein and its application in ZIKV vaccine development.

## Introduction

Zika virus (ZIKV) was first isolated in 1947 from a rhesus monkey taken from the Zika Forest in Uganda (Dick et al., 1952). Prior to the emergence of ZIKV epidemics, there had been only a few reported cases of ZIKV infection in humans in Africa and Asia, most of whom presented with mild clinical symptoms (Smithburn, 1952; Saluzzo et al., 1981; Adekolu-John and Fagbami, 1983). A major outbreak of ZIKV was reported on Yap Island in 2007, which infected almost 73% of the island’s inhabitants (Duffy et al., 2009). Other sporadic outbreaks have been reported in Southeast Asia (Kwong et al., 2013; Buathong et al., 2015), and there have been major epidemics in French Polynesia and the Americas in 2014 and 2015, respectively (ECDC, 2014; Hennessey et al., 2016).

Zika virus outbreaks from 2015 onward have been of major public health concern due to their associated clinical complications, such as Guillain-Barre syndrome in adults and neonatal microcephaly, suggesting mother to child transmission of the virus (Oehler et al., 2014; Krauer et al., 2017; Counotte et al., 2018). Owing to congenital brain abnormalities linked to ZIKV, the World Health Organization (WHO) declared an urgent need to fully understand the lifecycle of ZIKV and its infection dynamics in order to develop effective control methodologies, vaccines and therapeutic targets to prevent future outbreaks (WHO, 2016).

Zika virus is mainly transmitted to humans through mosquito-borne vectors (Vanlandingham et al., 2016; Zanluca and Dos Santos, 2016; Kauffman and Kramer, 2017), though some human-to-human transmission has also been reported (Song et al., 2017). Phylogenetic analyses of ZIKV identified African and Asian lineages, with the Asian lineage being responsible for the epidemics detected thus far (Zhu et al., 2016). These analyses have raised as yet unanswered questions. Firstly, given that it was initially identified almost 60 years ago, why is the virus only causing human epidemics now? Secondly, despite being initially isolated from Africa, why do ZIKV strains of the African lineage not cause epidemics? Thirdly, which viral factors are responsible for the severity of infection and subsequent congenital abnormalities? When the virus was first isolated, it was reported that it was non-virulent until its seventeenth passage in Swiss albino mice, suggesting that the virus may have to undergo several bouts of purifying selection to adapt to a new host (Dick, 1952). The fact that data on Africa lineages is lacking further complicates our understanding of this virus, with Wetsman suggesting that the widely used African strain MR766 may not be truly representative of the original isolate (Wetsman, 2017). Wetsman also indicated that Asian strains have a more pronounced effect on the expression of genes involved in DNA replication and repair in neural cells than African strains. A phylogenetic analysis of ZIKV strains responsible for recent epidemics in Asia has further suggested that they have undergone several amino acid substitutions during either circulation in hosts or vectors, giving rise to several biological phenotypes (Zhu et al., 2016).

Zika virus has biological similarities to other flaviviruses, such as West Nile virus, Japanese encephalitis virus (JEV) and dengue virus. ZIKV has an approximately 11 kb positive-stranded RNA genome, with untranslated regions flanking the 5′ and 3′ ends of its open reading frame. The genome is translated into structural proteins [capsid (C), precursor membrane (prM), and envelope (E)] and non-structural (NS) proteins (NS1, NS2A, NS2B, NS3, NS4A, NS4B, and NS5) [reviewed in (Lin et al., 2018)]. The NS proteins are mainly responsible for evasion of the host’s cellular innate immune response and for viral replication (Wu et al., 2017). The structural proteins are involved in the assembly of infectious virions and successful ingress and egress of host cells (Li et al., 2008). ZIKV virion assembly involves: (i) prM interaction with E protein in the endoplasmic reticulum; (ii) encapsulation of the RNA genome with C protein and coverage with a lipid bilayer containing a prM-E protein complex to form immature virions; and (iii) cleavage of prM protein into M protein by furin or furin-like protease in the *trans*-Golgi network before release of mature virions (Pierson and Diamond, 2012; Lin et al., 2018). The prM proteins protect E proteins from premature fusion in the low-pH conditions during transportation in the *trans*-Golgi network. Prior to virion release from the cell, cleavage of prM to M protein results in release of the *pr* peptide, allowing rearrangement of E proteins into homodimers and facilitating virion maturation. Recent studies have revealed that ZIKV has a similar structure to other known flaviviruses (Kostyuchenko et al., 2016; Sirohi et al., 2016). A conserved region of prM protein has been identified among flaviviruses that has been shown to be critical for viral assembly (Yoshii et al., 2012). Therefore, inhibiting the function of prM during the viral lifecycle may incapacitate ZIKV infectivity and pathogenicity. ZIKV prM and E proteins are being used as antibody-activating epitopes in most of the ZIKV vaccines currently undergoing clinical trials (WHO, 2018). This short review seeks to highlight recent advances in our understanding of the role of ZIKV prM protein in virus pathogenicity and its use in vaccine development.

## Pre-Epidemic and Epidemic ZIKV Strain prM Proteins and Their Pathogenicities

Zika virus has been categorized into pre-epidemic and epidemic strains through analyses of complete genomes or polyprotein sequences of isolates, most of which came from humans but some are from primates or mosquito vectors (Ramaiah et al., 2017). Pre-epidemic strains represent isolates associated with sporadic human infections in both Africa and Asia prior to 2007 (Saluzzo et al., 1981; Adekolu-John and Fagbami, 1983). Epidemic strains represent ZIKV isolates linked to human infections from outbreaks on Yap Island in 2007 to more recent outbreaks reported in the Americas (Duffy et al., 2009; Hennessey et al., 2016). Further bioinformatics analyses of these two viral categories have suggested that some amino acids may have been substituted in the epidemic viruses that have remained unaltered in pre-epidemic strains (Wang et al., 2016; Zhu et al., 2016; Yuan et al., 2017; Bos et al., 2018). A recent genomic analysis of ZIKV strains revealed two amino acid substitutions in ZIKV prM protein isolated from strains contributing to epidemics in 2007 compared to that of a pre-epidemic Asian strain isolated in 1966 (Zhu et al., 2016). When pre-epidemic African strains were compared with all other sequenced epidemic strains, nine amino acid substitutions were identified in ZIKV prM protein (Zhu et al., 2016). Another study found 10 amino acid substitutions between pre-epidemic and epidemic ZIKV prM proteins (Bos et al., 2018) (**Table 1**). These genomic variations might have been driven by ZIKV adopting an urban-based transmission cycle targeting humans as hosts rather than the original sylvatic mode of transmission that normally involves *Aedes* mosquitoes and non-human primates (Ramaiah et al., 2017). Adoption of a new transmission mode exerts a selection pressure on the viral genome to enable it to maintain its replication efficiency. Epidemic ZIKV strains, including those associated with neurovirulence, have been shown to belong to the Asian lineage (Faye et al., 2014; Bos et al., 2018). The change in target host to humans, and especially to naïve human Asian populations (rather than African), may have driven the amino acid substitutions and protein evolution of ZIKV prM proteins (Ramaiah et al., 2017).

**Table 1:** Summary of ZIKV prM amino acid substitutions.

Pre-epidemic amino acid residue	Epidemic amino acid residue	Amino acid position in ZIKV prM	Amino acid position in ZIKV polyprotein	Reference
I	V	3	161	Bos et al., 2018
S	N	17	175^a^	Yuan et al., 2017; Bos et al., 2018
K	E	21	179	Zhu et al., 2016; Bos et al., 2018
A	P	26	184	Zhu et al., 2016; Bos et al., 2018
V	M	31	189	Wang et al., 2016; Zhu et al., 2016; Bos et al., 2018
H	Y	35	193	Zhu et al., 2016; Bos et al., 2018
V	I	36	194	Bos et al., 2018
K	R	124	282	Bos et al., 2018
V	A	138	296	Bos et al., 2018
V	A	140	298	Bos et al., 2018


*^*a*^This substitution has been associated experimentally with neurovirulence in neonatal mice.*

Yuan et al. (2017) studied the effects of the commonly observed epidemic ZIKV amino acid substitutions in C, prM, E, NS1, and NS5 viral proteins to assess their roles in enhancing viral replication efficiency. They found that among all mutants examined, a serine to asparagine amino acid substitution (S139N) of ZIKV prM protein, which is observed in most human epidemic strains, exhibited the greatest neurovirulence in neonatal mice. On reversing the substitution from asparagine to serine (N139S) in the epidemic strain, N139S mutants were less neurovirulent than S139N. Moreover, when human neural progenitor cells (hNPC) were infected with either the S139N or N139S mutant, the S139N mutant elicited more cell death than the N139S mutant or pre-epidemic Asian wild type virus. These findings suggest that this serine to asparagine amino acid substitution in epidemic strains may have contributed to the recently observed congenital birth defects associated with ZIKV outbreaks in Brazil (Krauer et al., 2017). The contributions of other prM substitutions to ZIKV neurovirulence remain to be studied in depth as Yuan et al. (2017) studied only two amino acid substitutions in prM. More work is needed to identify other genetic factors contributing to pathogenesis since the N139S reverse substitution only reduced neurovirulence and did not completely prevent it.

Another study suggested that the highly thermostable ZIKV E protein, unlike DENV E protein, may also be associated with neurovirulence (Kostyuchenko et al., 2016). The surface of DENV2 virus changed structurally when the virus was incubated at 37°C, whereas ZIKV virus particles remained structurally stable, most likely due to the tightly packed and complex interactions of ZIKV E dimers. The ability of ZIKV to survive harsh conditions may explain why the virus remains viable in different body fluids such as semen, saliva and urine (Gourinat et al., 2015; Musso et al., 2015; Atkinson et al., 2016). The relative contributions of prM amino acid substitutions to E protein thermostability and consequent ZIKV structural biology are not yet well known. However, changes in prM protein structure may induce structural changes in E protein as E protein assembly is dependent on prM protein expression (Oliveira et al., 2017). As the virus undergoes purifying selection in the environment, prM modifications might be responsible for the production of novel infectious viral particles that enhance virulence in the host, but this hypothesis needs to be empirically tested.

## Perspectives for the Use of prM in ZIKV Vaccine Development

The ZIKV viral envelope is comprised of two viral proteins, the prM/M and E proteins. Since prM is cleaved to M during virion maturation, prM is usually not present on the virions or at a very low levels. Although M proteins are protected by E proteins in mature virions, both prM and E proteins have become major targets of vaccine design and development against ZIKV infections (**Table 2**) (WHO, 2018). These structural proteins have been targeted because they are: (i) non-replicating subunits of the ZIKV genome so they present a safer candidate profile for vaccines; (ii) the main determinants for the high stability of ZIKV; and (iii) epitopes for CD4^+^ and CD8^+^ *T*-cell adaptive immune responses and neutralizing antibodies (unlike other flaviviruses such as dengue virus whose *T*-cell epitopes are located in the non-structural proteins) (Chahal et al., 2017; Grifoni et al., 2017; Goo et al., 2018).

**Table 2:** ZIKV vaccine candidates undergoing clinical trials^a^.

Sponsor/Developer	Platform	Immunogen	Adjuvant	Phase	Reference for published results
GeneOne/Inovio	DNA	prM-E	None	Phase 1	Tebas et al., 2017
GeneOne/Inovio	DNA	prM-E	None	Phase 1	Not available
NIH	Peptide	mosquito salivary proteins	ISA-51	Phase 1	Not available
Themis bioscience	Recombinant viral vector	prM-E	None	Phase 1	Not available
Moderna therapeutics	mRNA	prM-E	None	Phase 2	Not available
NIAID	DNA	prM-E	None	Phase 1	Gaudinski et al., 2018
NIAID	DNA	prM-E	None	Phase 1	Gaudinski et al., 2018
NIAID	DNA	prM-E	None	Phase 2	Not available
NIAID	Inactivated virus	whole virus	Alum	Phase 1	Modjarrad et al., 2018
NIAID	Inactivated virus	whole virus	Alum	Phase 1	Modjarrad et al., 2018
BIDMC	Inactivated virus	whole virus	Alum	Phase 1	Modjarrad et al., 2018
NIAID	Inactivated virus	whole virus	Alum	Phase 1	Not available
Takeda	Inactivated virus	whole virus	Alum	Phase 1	Not available
Valneva austria GmbH	Inactivated virus	whole virus	Alum	Phase 1	Not available


*^*a*^ZIKV vaccine candidates undergoing clinical trials according to the World Health Organization (WHO, 2018).*

The ZIKV vaccines currently under development are mainly based on purified inactivated viruses, plasmid DNA, or mRNA platforms (Tebas et al., 2017; Gaudinski et al., 2018; Modjarrad et al., 2018). Importantly, none of these ZIKV vaccine approaches have achieved success in terms of generating highly effective neutralizing antibodies by using prM or E protein alone. Instead, a combination of prM-E structural proteins is required, even if the *pr* peptide of prM protein might not be present in the final vaccine formulation.

### Plasmid DNA-Based Vaccines

Larocca et al. (2016) demonstrated that a prM-E DNA vaccine could provide complete protection in mice against challenge with ZIKV strains linked to major clinical complications. That study also noted that a prM-deleted mutant plasmid DNA vaccine could not offer the same protection, evidencing the important role of prM protein in enhancing ZIKV immunogenicity. The ZIKV prM-E DNA vaccine resulted in a higher titer of envelope antibody production as well as ZIKV-specific neutralizing antibodies but not prM-specific antibodies. This phenomenon has also been reported in another study using a ZIKV prM-E immunogen as a vaccine candidate (Yi et al., 2017). This outcome may be due to the structural nature of ZIKV E protein as it is the main viral protein participating in binding and fusion to host receptors, enabling easy access to this epitope by host immunity. Although the antibody-recognizing epitope is located on the E protein, prM is suggested to enhance immunogenicity because prM interacts with domain II of E protein to avoid premature virion release (Pierson and Diamond, 2012; Kostyuchenko et al., 2016; Oliveira et al., 2017). Other studies have also reported that ZIKV-neutralizing antibodies do not bind to fully mature virions but only to immature virions (Dai et al., 2016), and the prM proteins of these latter may be processed during ingress by furin proteases available in endocytic vesicles (Pierson and Diamond, 2012). Another DNA vaccine encoding full-length prM-E protein has also shown the ability to confer full protection against ZIKV challenge in humanized DRAG mice that have robust antibody responses and are useful for testing vaccination approaches (Danner et al., 2011; Yi et al., 2017).

### Vaccines Based on Inactivated Viruses or Virus-Like Particles (VLP)

A vaccine produced by immunizing mice with VLPs that incorporates full-length ZIKV structural proteins (C-prM-E) can also endow full protection against ZIKV and induces production of more ZIKV-neutralizing antibodies than DNA vaccines (Garg et al., 2017). A chimeric live-attenuated ZIKV prM-E vaccine candidate, using a commercialized JEV vaccine as a backbone (Li et al., 2018), has also demonstrated robust immunization and complete protection in both rhesus macaques and AG129 mice that present a receptor deficiency for interferon (IFN)-α/β and IFN-γ (Lazear et al., 2016). The JEV prM-E was replaced with the corresponding prM-E from an Asian ZIKV strain. Another approach using chimeric ZIKV with DENV prM-E proteins or a chimeric DENV with ZIKV prM-E proteins revealed robust protection in immunized mice through the production of highly neutralizing antibodies (Xie et al., 2017). The main concern with such vaccines is whether they will result in the emergence of naturally circulating genetically modified infectious viruses.

### RNA-Based Vaccines

Successful immunization and neutralizing antibody production in C57BL/6 mice has been demonstrated for an RNA nanoparticle vaccine based on prM-E (Chahal et al., 2017). This vaccine uses an RNA replicon vector to package ZIKV prM-E protein for expression in BHK21 cells. Protein expression results in the release of small viral particles (SVPs) that exhibit similar functions to fully functional virions but are smaller than the wild type virions that contain capsid proteins (Chang et al., 2001; Chahal et al., 2017). The replicon RNA can then be used to form a modified dendrimer nanoparticle (MDNP) RNA vaccine.

Modified mRNA vaccines based on ZIKV prM-E proteins have also been developed (Pardi et al., 2017). These modified mRNA vaccines present a next generation vaccine platform. This approach involves encoding wild type prM-E genes in an mRNA with a nucleoside 1-methylpseudouridine modification to avoid sensing by the host innate immune system. The modified mRNAs encoding ZIKV prM-E proteins are then encapsulated in lipid nanoparticles for efficient protein expression (Pardi et al., 2017, 2018; Richner et al., 2017). The mRNAs are then expressed in either HK293T or HeLa cells, resulting in the production of SVPs that can induce production of neutralizing antibodies. These modified ZIKV mRNA vaccines use non-self-amplifying mRNA that cannot integrate into the genome, unlike DNA vaccines. The vectors used are also non-infectious and just a small dose can induce effective immunization (Pardi et al., 2018). These mRNA vaccines have exhibited robust immunization and protection against ZIKV infection in both immunocompromised AG129 and C56BL/6 mice. ZIKV mRNA vaccine concepts are currently undergoing clinical trials and success would prove to be an important milestone in combating current and future ZIKV outbreaks.

## Concluding Remarks

Zika virus has become a major public health concern, with outbreaks of the disease spreading across the globe and resulting in congenital birth defects in some instances. Evolutionary and purifying selection pressures might have facilitated modifications of the ZIKV genome that enhance its replication efficiency and reduce host immune efficiency as it adapted to new hosts in urban environments. This has led to virus strains being categorized as either pre-epidemic or epidemic, with the latter being associated with recent infection outbreaks. Thus far, the S139N amino acid substitution of prM protein has been linked to increased neurovirulence, suggesting that alterations of ZIKV prM protein may enhance viral virulence. Further studies on other observed amino acid substitutions in all viral proteins should be carried out to establish their roles in viral replication efficiency.

Various platforms have been employed to generate vaccines against ZIKV infections, including purified inactivated virus, plasmid DNA, and mRNA nanoparticles. Despite these platforms having been adapted from studies of other flaviviruses, the resulting vaccines have exhibited experimental success when a combination of both prM and E proteins are utilized. In contrast, sole use of ZIKV prM protein in vaccines against ZIKV has not yet proven as effective as prM-E combinations.

It remains unclear if future epidemics will have the same clinical implications as the most recent epidemic or which viral protein modifications are more likely to result in another epidemic. Furthermore, whether the ZIKV vaccines currently under development can be effective against future epidemics is uncertain. These basic questions should guide research efforts for the prevention, control and therapeutic strategies of ZIKV. Additional focused studies on the molecular and structural biology of ZIKV prM may accelerate our ability to tackle this emerging public health concern.

## Author Contributions

PN wrote the manuscript. W-CS contributed to the framework and the editing of the manuscript.

## Conflict of Interest Statement

The authors declare that the research was conducted in the absence of any commercial or financial relationships that could be construed as a potential conflict of interest.
